# Calibration for Quantitative Chemical Analysis in IR Microscopic Imaging

**DOI:** 10.1021/acs.analchem.5c03049

**Published:** 2025-10-06

**Authors:** Eirik Almklov Magnussen, Boris Zimmermann, Simona Dzurendova, Ondrej Slany, Valeria Tafintseva, Kristian Hovde Liland, Kristin To̷ndel, Volha Shapaval, Achim Kohler

**Affiliations:** † Faculty of Science and Technology, 56625 Norwegian University of Life Sciences, Ås, Akershus 1432, Norway; ‡ Faculty of Chemistry, 48274 Brno University of Technology, Brno 60190, South Moravia, Czech Republic

## Abstract

Infrared spectroscopy of macroscopic samples can be calibrated against reference analysis, such as lipid profiles acquired by gas chromatography, and serve as a fast, low-cost, quantitative analytical method. Calibration of infrared microspectroscopic images against reference data is in general not feasible, and thus spatially resolved quantitative analysis from infrared spectral data has not been possible so far. In this work, we present a deep learning-based calibration transfer method to adapt regression models established for macroscopic infrared spectroscopic data to apply to microscopic pixel spectra of hyperspectral IR images. The calibration transfer is accomplished by transferring microspectroscopic infrared spectra to the domain of macroscopic spectra, which enables the use of models obtained for bulk measurements. This allows us to perform quantitative chemical analysis in the imaging domain based on infrared microspectroscopic measurements. We validate the suggested microcalibration approach on microspectroscopic data of oleaginous filamentous fungi, which is calibrated toward lipid profiles obtained by gas chromatography and measurements of glucosamine content to perform quantitative infrared microspectroscopy.

## Graphical abstract

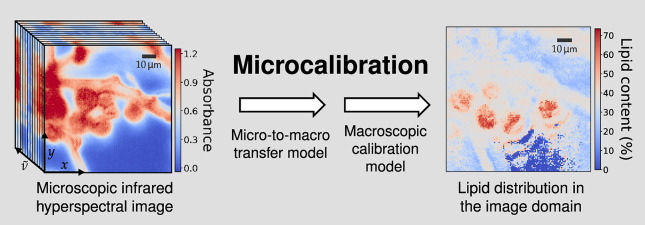

## Introduction

Infrared (IR) spectroscopy is a well-established, nondestructive, and label-free technique for probing the chemical composition of samples quickly and efficiently.^1–3^ In its macroscopic mode, IR spectroscopy can be used to analyze the bulk chemical composition, either through conventional single-spectrum setups or using high-throughput screening (HTS) extensions.^4^ In microscopic mode, an IR microscope is employed to yield spatially resolved analysis of cells and tissues, providing detailed information on molecular absorption at the microscale. IR hyperspectral imaging has become a fairly inexpensive and fast method which delivers extensive data on the spatial distribution of molecular absorption and simultaneously reveals physical properties such as refractive index and sample morphology.

To enable quantitative analysis using bulk IR spectra, calibration models must be developed by correlating spectral data with chemical reference analyses. Thus, the calibration models infer the result of chemical analysis from the IR spectra. This approach has been successfully applied in numerous fields,^5,6^ offering the advantage of low-cost, automated, and rapid analysis both in laboratory and field environments. This has led to widespread use in industries like food and feed quality control,^7^ dairy breeding programs,^8^ soil science,^9^ water monitoring,^10^ and measuring hydrolysates^6^ and proteins.^11^ Furthermore, the rising demand for lipids in fuel, food, and feed has led to increased focus on their industrial production and extraction. Calibration models for lipid profiles and metabolites based on IR spectra that were calibrated against gas chromatography (GC) and high-performance liquid chromatography (HPLC), are now used to monitor extraction processes, assess lipid accumulation, and identify key fatty acids.^12–15^ These models can streamline biorefineries and other industrial processes by reducing the need for expensive and time-consuming downstream chemical analyses.

While the use of IR spectroscopy and calibration models for quantitatively assessing bulk chemical composition has become nearly standard practice, developing calibration models for microscopic imaging data remains a significant challenge. Although acquiring IR hyperspectral images is relatively straightforward, the lack of spatially resolved reference data makes quantitative analysis difficult. Consequently, IR imaging is most commonly applied to segmentation and identification^16–18^ rather than quantification, and establishing calibration models for IR images of intact cells and tissues has generally been deemed unfeasible.

A technology that enables true quantitative, spatially resolved mapping of chemical distributions in cells and tissues could profoundly impact and advance the field of biological research. This advancement would pinpoint the exact locations where various chemical compounds are created and stored in microorganisms. As a result, it would provide detailed quantitative insights into cellular biochemical processes and rapid access to the molecular composition throughout the cells and tissues studied.

In the following, we propose a microcalibration approach that bridges the gap between macroscopic infrared spectra and microscopic IR hyperspectral images. This enables the transfer of calibrations from bulk IR measurements to the imaging domain, facilitating the application of these models to hyperspectral imaging data. While conventional calibration transfer is a well-known technique for instruments that measure biomass in similar ways,^19–22^ transferring calibration from macroscopic spectra to microscopic hyperspectral images is significantly more complex due to the distinct measurement techniques and the lack of a one-to-one relationship between the instruments and treatments of the biomass.

Two key obstacles must be overcome to enable successful calibration transfer from bulk to spatially resolved IR measurements. First, Mie-type scattering is a pervasive issue in infrared microscopy of intact cells and tissues and must be effectively addressed. Scattering in the mid-infrared range is governed by the sample’s morphological, chemical, and optical properties, resulting in a high interdependence between absorption and scattering signals.^23^ Since calibration models typically rely on absorption signals, it is crucial to separate the scattering and absorption components in scatter-distorted spectra. This challenge is especially critical in microscopy, as scattering effects are more pronounced in IR microscopy of intact microsamples compared to bulk measurements. To address this, we combine electromagnetic theory with machine learning to separate scattering and absorption signals in distorted spectra. Previous work has demonstrated promising results toward separation of these signals in measured spectra using deep learning models,^24–26^ providing a pathway to obtain scatter-free hyperspectral images suitable for calibration transfer.

Second, macroscopic bulk and microscopic imaging measurements are, although they rely on the same physical processes, different techniques. They each have unique optical configurations, including objectives, detectors and illuminations, which yield distinct spectral results. In addition to using different instruments, sampling scales, spatial resolutions and sample treatments, these methods also differ in dimensionality: hyperspectral images are two-dimensional, while bulk measurements are one-dimensional. On average, an image pixel spectrum should chemically resemble the bulk spectrum of the same sample. However, a single pixel spectrum does not directly correspond to any specific bulk measurement spectrum. This disparity creates a challenge in developing calibration transfer models that can bridge the gap between bulk and imaging measurements. To address this, we propose a solution in which homogenized biomass is first measured with an imaging system, followed by a bulk measurement of the same sample. This ensures that each pixel spectrum and the bulk measurement for any given sample correspond to the same biomass, enabling the development of a calibration transfer model that reconciles the differences between bulk and spatially resolved IR measurements. This transfer model should account for the difference in instrumentation, dimensionality and the optical configuration.

We believe that by combining electromagnetic modeling with deep learning, along with meticulous data collection and experimentation, calibration transfer can be achieved. This will enable microcalibration of infrared hyperspectral images of intact cells and tissues. Our method uses regression models based on macroscopic HTS-FTIR data to predict chemical concentrations in FTIR hyperspectral images. We validate this approach by quantifying lipid content, fatty acid profiles (saturated and polyunsaturated), and glucosamine in filamentous fungi grown under various conditions. We demonstrate the ability to predict the spatially resolved lipid and glucosamine content in biological samples. This innovative method provides a cost-effective tool for rapid, spatially resolved chemical analysis, yielding insights that are difficult to obtain using traditional methods like GC. The potential applications in both research and industry are vast, offering new opportunities for studying the biochemistry of biological cells in unprecedented detail.

## Materials and Methods

The microcalibration model consists of two individually trained models as shown in Figure 1, namely a regression model and a transfer model. To perform microcalibration we subsequently apply the transfer model and the regression model to every pixel of a hyperspectral image. The regression model is trained to infer the result of the reference analysis, such as gas chromatography, of a sample based on macroscopic bulk IR measurement, such as an HTS-FTIR spectrum. This is a conventional calibration model trained on annotated data, where the model learns from the GC reference analysis to infer the lipid profile when provided with HTS-FTIR spectra as input. We establish a transfer model which accounts for the inherent variability between the pixel spectra of a microspectroscopic image and the macroscopic HTS-FTIR spectra of the same biomass. All of the intervariability between microscopic and macroscopic measurements should be accounted for by the transfer model. Thus, the transfer model should handle the difference in both optics and instrumentation, as well as the fact that the light-matter interaction manifests itself differently in bulk spectroscopic measurements, since the wavelength is much shorter than the sample sizes in macroscopic measurements. The model must effectively handle the light scattering, as this is prominent in microspectroscopy and generally less so in macroscopic measurements.

**Figure 1:**
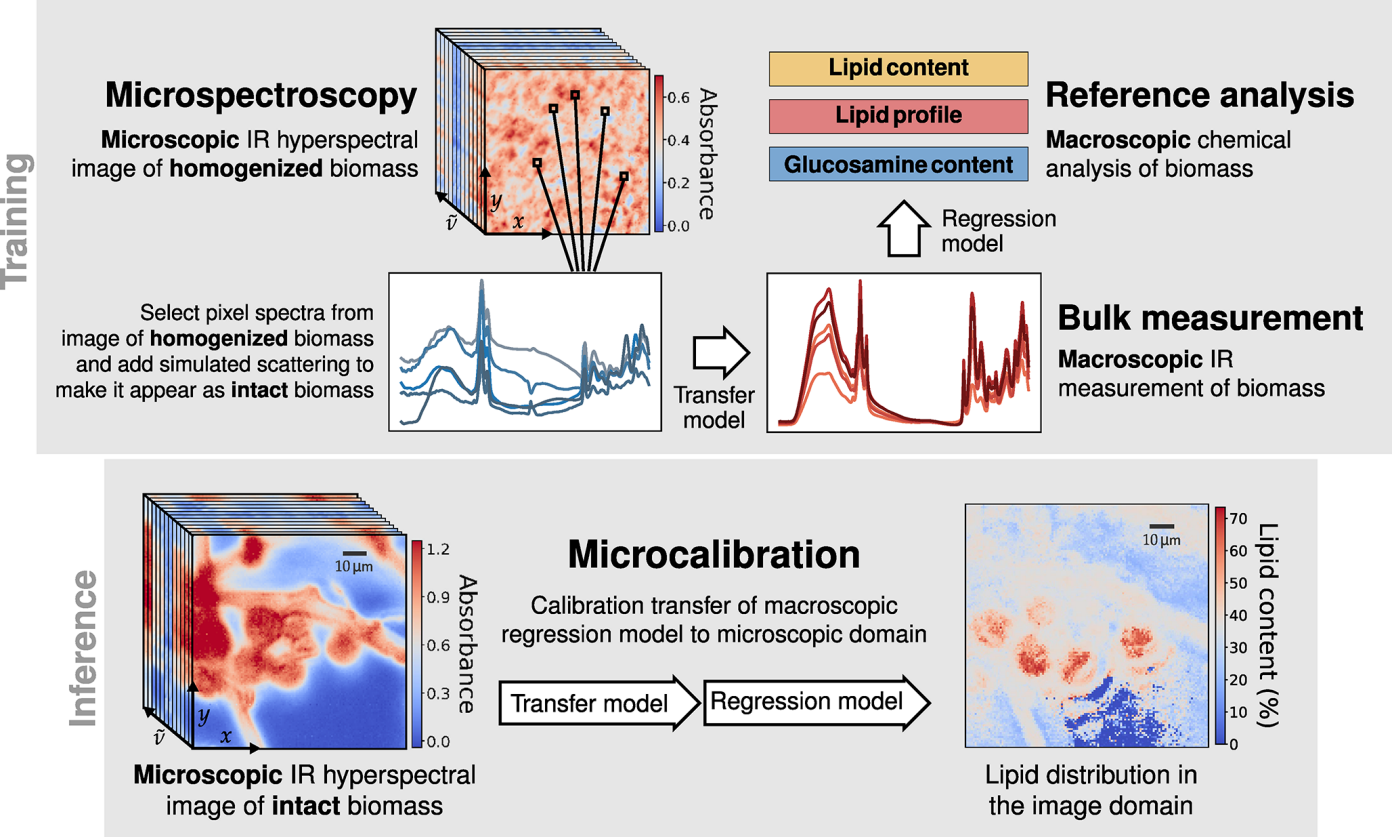
Illustration of the suggested approach for establishing a microcalibration model. We train a regression model to infer the result of reference analysis such as GC or glucosamine measurements from macroscopic HTS-FTIR spectra. Further, we build a transfer model from microspectroscopic data of homogenized biomass and macroscopic spectra of the same samples, which accounts for the variability between the microscopic and the macroscopic FTIR measurements. Finally, microcalibration is achieved by sequential application of the transfer and the regression model on microspectroscopic images of intact biomass.

We have procured a data set containing both GC reference analyses and macroscopic IR spectroscopic measurements, as well as microscopic measurements of both intact and homogenized biomass. Having all these measurements available for each sample in the data set, enables us in building the microcalibration model. For biologically identical samples we have macroscopic HTS-FTIR measurements of the biomass and IR microspectroscopic hyperspectral images of both intact and homogenized biomass, as well as GC analysis of the lipid content and profile. We can use macroscopic IR spectral measurement of the bulk biomass and the microscopic hyperspectral images of homogenized biomass to build a transfer model, which accounts for the difference between macroscopic and microscopic measurements. This can be done since the difference between the pixel spectra of the image of homogenized biomass and macroscopic spectra should largely represent the identical chemical composition. We can further use this to establish a microcalibration model which infers the spatial distribution of the lipid profile from a microspectroscopic hyperspectral image. To do this the transfer model transfers the measured imaging data such that we can use calibration models established for macroscopic spectra on the pixel spectra of the images.

Furthermore, we have a data set containing HTS-FTIR spectra and measurements of the glucosamine content for 48 samples of one species of filamentous fungi. This will be used to indicate that we can apply the method to infer the spatially resolved glucosamine content and demonstrate that the microcalibration approach is valid for various types of reference analysis.

Additionally, we have a separate data set comprising 16 FTIR microspectroscopic measurements of *Mucor circinelloides* filamentous fungi. The samples were cultivated in media with varying inorganic phosphate salt concentrations, and the acquisition of the IR images is described in Magnussen et al.^25^ It will be used for independent qualitative validation on a data set not at any stage used to establish neither the calibration transfer model nor the regression models.

### Sample Preparation

The main data set used in the following, consists of 36 distinct samples of oleaginous filamentous Mucoromycota fungi. Six fungal strains of five different species were cultivated under different growth conditions. We have strains of the species *Mucor circinelloides* (two distinct strains), *Amylomyces rouxii*, *Rhizopus stolonifer*, *Umbelopsis vinacea*, and *Mucor racemosus* which have been grown in media containing three different concentrations of phosphorus (0.5×, 1× and 4× the reference amount) and with or without calcium supplementation. The species belong to four different genera (*Amylomyces*, *Mucor*, *Rhizopus*, and *Umbelopsis*) and two different families (Umbelopsidaceae and Mucoraceae), which ensure that we have a high biochemical diversity in our data set. Further details on the fungal cultivation and sample preparation can be found in Dzurendová et al.^15^

### Reference Analysis

For the 36 samples in the main data set the lipid extraction and determination of fatty acid profiles and lipid concentration using gas chromatography (GC) with flame ionization detection analysis was performed as described in Dzurendová et al.^15^

We measured the glucosamine (GlcN) content of 48 distinct samples. Determination of GlcN content requires first a preparation of alkali insoluble material (AIM). AIM was prepared using 0.5 mol/L sodium hydroxide (NaOH) solution. Tested samples (10–100 mg) were placed into 10 mL screw-cap tubes and mixed with 3 mL of 0.5 mol/L NaOH solution. Tubes were placed into thermoheater and incubated for 18 h at 90 °C. Subsequently, samples were cooled down to 25 °C and centrifuged (5000 rpm, 10 min). Supernatants were removed, and solid layer was washed with distilled water (5 × 5 mL). Obtained purified AIMs were freeze-dried for 72 h. Dry AIMs were weighted and stored in the freezer (−23 °C) for further analysis. To measure the GlcN content, samples of AIM (5–25 mg) were placed in 10 mL screw cap tubes and mixed with 5 mL of 6 hydrochloric acid. Tubes were incubated at 100 °C for 12 h. Afterward, samples were cooled down to 25 °C and processed according to the method presented by Aidoo et al.^27^ with minor modifications according to Slany et al.^28^ Then 0.5 mL of supernatant was placed into 25 mL volumetric flask and mixed with 5 μL of phenolphthalein solution (1% EtOH solution). The solution was titrated by NaOH solution (1 mol/L) until color was changed to purple, and immediately titrated back with KHSO_4_ 1% w/w) until colorless solution was obtained. Final volume was adjusted to 25 mL with distilled water. Further, 1 mL of such solution was placed into 20 mL glass tube and mixed with NaNO_2_ solution (1 mL; 5% w/w) and KHSO_4_ solution (1 mL; 5% w/w). Reaction tubes were mixed and incubated at 25 °C for 15 min. Afterward, ammonium sulphamate solution was added (1 mL; 12.5% w/w), tubes were mixed and incubated another 15 min. MBTH solution (1 mL; 0.5% w/w) was added and tubes were immediately incubated at 100 °C for 15 min. Afterward, reaction tubes were cooled down to 25 °C and samples were mixed with FeCl_3_ solution (1 mL; 0.83% w/w). Samples were incubated at 25 °C for 45 min and absorbance of obtained blue-colored product was measured at 650 nm against control (blank) sample that was prepared in the same conditions as the actual test sample, except that distilled water was used instead of the sample supernatant. Amount of GlcN units was quantified by calculation with the standard calibration curve. All experiments were performed in three independent technical replicates.

### IR Spectroscopic Measurements

IR spectroscopy works by measuring the loss of radiation as a function of wavenumber ν̃, as encoded in the absorbance *A*(ν̃). Beer–Lambert law states that

$$A(\tilde{\nu })=l\sum_{i}C_{i}\epsilon_{i}(\tilde{\nu })$$ 1

where ϵ_*i*_ are molar absorption coefficients, $C_{i}$ molar concentrations and *l* the optical path length. That is, in the ideal case, any macroscopic or microscopic IR measurement reveal information about concentrations of chemical agents in the sample by considering the peak heights of peaks corresponding to the chemical agents in question.

For the different strains of the fungi grown under varying conditions we have for each of the 36 samples a microscopic FPA hyperspectral image of both homogenized and intact biomass. For the very same fungal strains, we obtained macroscopic spectra of cell populations using a high throughput system measurement (HTS-FTIR) as well as the GC reference analysis. The samples were grown under the exactly same conditions and samples were prepared in three independent biological replicates. For homogenization, approximately 5 mg of biomass was transferred into a 2 mL polypropylene tube containing (250 ± 30) mg of acid-washed glass beads and 0.5 mL of distilled water. The homogenization of fungal biomass was performed using a Precellys Evolution tissue homogenizer (Bertin Technologies, France) working at 5500 rpm on a 6 × 20 s cycle. For measurements 10 μL of homogenized fungal biomass was pipetted onto a silicon IR transparent microplate for HTS-FTIR or a ZnSe window for FTIR microspectroscopy.

The FTIR-HTS transmittance spectra were measured using a High Throughput Screening eXTension (HTS-XT) unit coupled to a Vertex 70 FTIR spectrometer (both Bruker Optik, Germany), equipped with a globar mid-IR source and a deuterated triglycine sulfate (DTGS) detector. 10 μL of either supernatant or homogenized biomass was pipetted onto an IR transparent 384-well silica microplate, and dried at room temperature for 2 h. The HTS-FTIR spectra were recorded with a total of 64 scans, spectral resolution of 6 cm^–1^, and digital spacing of 1.928 cm^–1^, over the range of 4000–400 cm^–1^, and an aperture of 5 mm. Spectra were recorded as the ratio of the sample spectrum to the spectrum of the empty IR transparent microplate. Each sample was analyzed in three technical replicates.

The microscopic infrared data used in the following consist of 36 microspectroscopic hyperspectral images of both intact as well as homogenized biomass of each of the oleaginous fungal strains. The FTIR microspectroscopy transmittance spectral images were measured using a Hyperion 3000 IR microscope coupled to the Vertex 70 FTIR spectrometer (both Bruker Optik, Germany), equipped with a globar mid-IR source, a liquid nitrogen-cooled mercury cadmium telluride (MCT) 128 × 128 focal plane array (FPA) detector system, and a computer-controlled *x*/*y*/*z* stage. For the measurement of intact samples, approximately 1–2 mg of washed biomass was resuspended in 0.5 mL of distilled water, and deposited onto a 1 mm thick and 25 mm diameter IR transparent ZnSe window, and dried at room temperature for 2 h. For homogenized samples, the aforementioned homogenized fungal biomass was pipetted onto the ZnSe window, and dried at room temperature for 2 h. The spectra were recorded with a total of 128 scans in the 3845–900 cm^–1^ spectral range, with a spectral resolution of 8 cm^–1^, and digital spacing of 3.8522 cm^–1^. The samples were measured using a × 15 objective and numerical aperture of 0.4. Background spectra were recorded at the start of each measurement by measuring a sample-free area of the ZnSe slide. For each intact sample, four hyperspectral tiles (128 × 128 pixels each) were recorded, resulting in composite (2 × 2 tiles, 256 × 256 pixels) hyperspectral images. For each homogenized sample, one hyperspectral tile was recorded.

The OPUS 8.2 software (Bruker Optik GmbH, Germany) was used for data acquisition and instrument control for both microscopic and macroscopic measurements. We finally interpolate the macroscopic spectra to share the wavenumber range of the microscopic pixel spectra.

### Mie Scattering

IR microspectroscopy of intact cells and tissue generally is distorted by highly nonlinear electromagnetic scattering. This needs to be accounted for in order to establish a transfer model that is applicable to intact samples. Mie-type scattering is light scattering on spherically shaped samples. However, we have shown that theoretical Mie models are as well applicable for particles that differ in their form considerably from a sphere.^29^ For a sphere of radius *r* and complex refractive index *n*, the presence of Mie scattering invalidates Beer–Lambert law’s assumptions such that we have nonexponential attenuation.^30,31^ The absorbance becomes

$$A(\tilde{\nu })=-\log_{10}\left(1-\frac{g_{\mathrm{NA}}}{G}Q_{\mathrm{ext}}^{\mathrm{NA}}\right)$$ 2

where *g*_NA_ and *G* represent the cross-sectional area of the detector’s effective numerical aperture (NA) and the sample respectively, and *Q*_ext_^NA^ is the extinction efficiency, which quantifies how much radiation is removed from hitting into the numerical aperture by the sample. The extinction efficiency is a highly nonlinear function of wavenumber and can be written as a linear combination of the scattering efficiency away from the NA and the absorption efficiency as

$$Q_{\mathrm{ext}}^{\mathrm{NA}}=cQ_{\mathrm{abs}}+Q_{\mathrm{sca}}^{\mathrm{NA}}$$ 3

The scattering efficiency for a sphere is given by the diagonal elements of the scattering amplitude matrix S as^32^

$$Q_{\mathrm{sca}}^{\mathrm{NA}}=\frac{1}{2(\pi r\tilde{\nu }{)}^{2}}\int_{\theta_{\mathrm{NA}}}^{\pi }(|S_{1}{|}^{2}+|S_{2}{|}^{2})\sin\;\theta d\theta$$ 4

Mie scattering is particularly pronounced when the wavelength of the radiation is on the same length scale as the sample size, which is the case for IR radiation and many biological cells, which both are on a micrometre scale.^23^ Using neural networks for correcting scatter-distorted IR spectra have been shown to work very well.^24,25^ We augment the homogenized microspectroscopic data set by adding simulated Mie scattering to the spectra, illustrated in Figure 2c. The simulations cover a range of radii (*r* ∈ [2 μm, 18 μm]) and constant, real parts of refractive indices (*n*_0_ ∈ [1.2, 1.65]), corresponding to varying morphologies and optical properties. The complex refractive index is *n* = *n*_0_ + *n*_*r*_(ν̃) + *in*_*i*_(ν̃), where *n*_*i*_ is proportional to scatter-free absorbance spectra and *n*_*r*_ and *n*_*i*_ are related through a Kramers–Kronig relation.^25,32^ Thus, we let our transfer model learn to correct for the scatter-distortion. The scattering simulation follows Magnussen et al.,^25^ but we use a smaller effective numerical aperture θ_NA_ for the detector to reflect the pixel-level resolution of microspectroscopic measurements. Given that our setup uses an aperture-free detector, the effective aperture is determined by the pixel size, and the smaller effective numerical aperture is therefore appropriate. We also augment spectra with polynomial baselines and Gaussian white noise.

**Figure 2:**
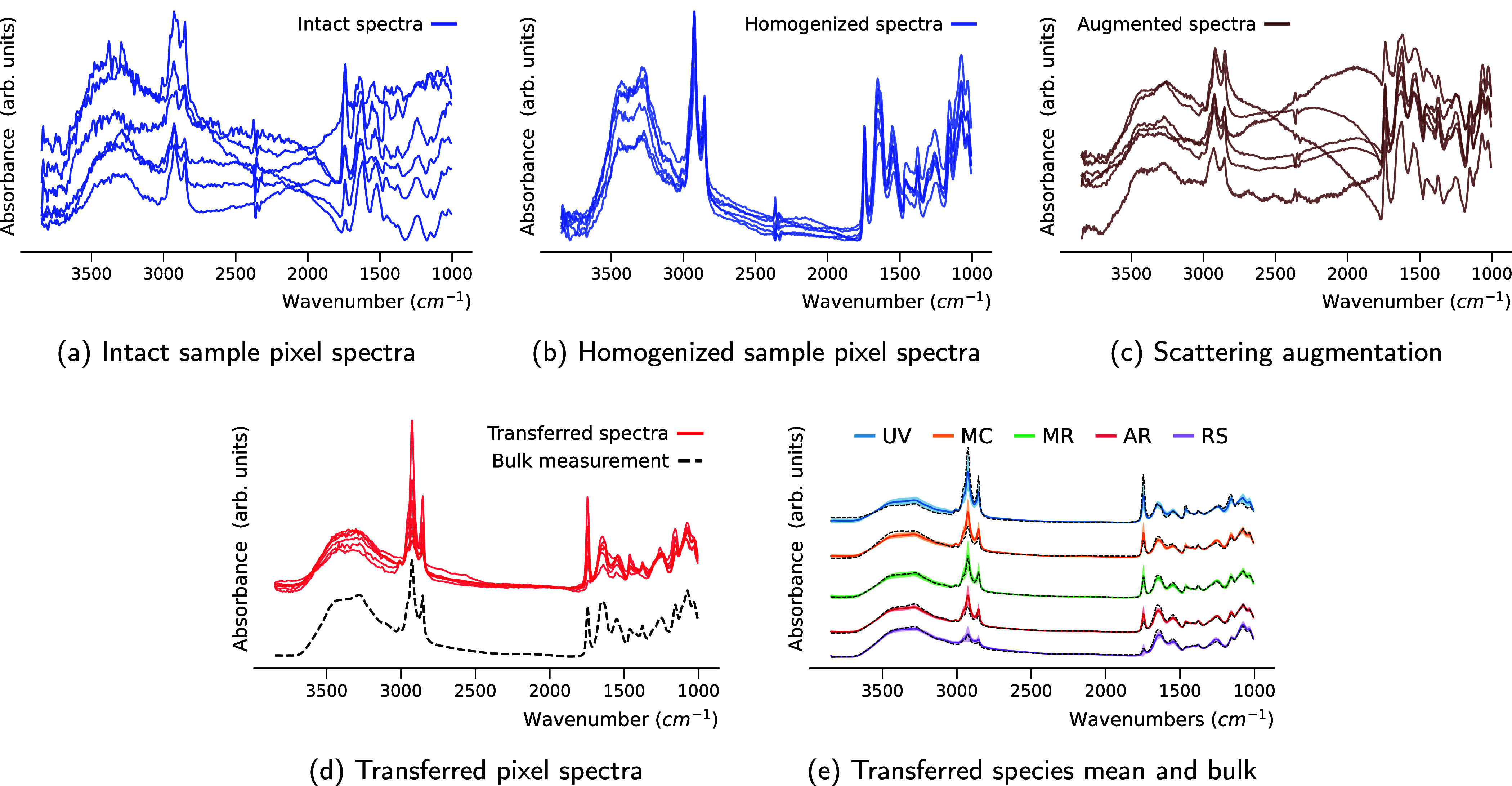
(a) Scatter-distorted pixel spectra from single unprocessed hyperspectral image of the intact biomass of a *Mucor racemosus* fungal strain. (b) Unprocessed microspectroscopic spectra of homogenized biomass of the same strain. (c) Homogenized spectra after augmentation with simulated Mie scattering (d) Transferred spectra of intact biomass, shown together with the corresponding bulk (HTS-FTIR) spectrum of the same strain. (e) Mean pixel spectrum and standard deviation for transferred hyperspectral images of one fungal strain per species *Mucor circinelloides* (MC), *Amylomyces rouxii* (AR), *Rhizopus stolonifer* (RS), *Umbelopsis vinacea* (UV) and *Mucor racemosus* (MR) cultivated under the same growth conditions. Stipulated black lines indicate the corresponding HTS-FTIR spectra.

### Establishing the Microcalibration Model

We start by training conventional regression models to perform calibration. The models are deep attention-augmented convolutional neural network^33^ trained to infer the result of the reference analysis of a sample from the HTS-FTIR spectrum. We build one model for inferring each of the quantities under consideration, namely the lipid content, the percentage of fatty acids which are SFA and PFA, and the concentration of glucosamines.

In order to establish a transfer model from microscopic to the macroscopic domain, we train another attention-augmented convolutional neural network to be able to account for the difference between HTS-FTIR and FPA microspectroscopic measurements of the same samples. To this end we randomly select pixel spectra from the microspectroscopic image of homogenized biomass and let the model infer the HTS-FTIR spectrum of the corresponding biomass. We make the assumption that on average the pixel spectra in the homogenized hyperspectral image correspond to the same molecular information as the one encoded in the corresponding HTS-FTIR spectrum. Although the homogenization process works fairly well, it is not flawless, and there can be some small regions in the image with either nearly no sample or clusters of one particular type of biomass such as lipid bodies produced by the fungi. To handle this we exclude pixel spectra with very poor signal and those which deviated too much from the mean spectrum within any given hyperspectral image. This ensures that the macroscopic and the microscopic pixel-spectra of homogenized biomass should largely represent the same molecular composition, such that the main difference between the spectra stems from the inherent difference between a pixel spectrum collected in an imaging system and a macroscopic spectrum. This difference can emerge due to variations in the instrumentation, detector, numerical aperture, light source, optics and sample holder. However, many of these differences arise from light-matter interactions that behave distinctively in microscopic environments, particularly due to the effects of nonlinear light scattering. Both calibration and transfer models are trained using RMSProp optimizer minimizing the mean-squared error loss. Calibration models train in minutes, but transfer models, being more complex, require several hours of training.

## Results and Discussion

Figure 2 shows examples of pixel spectra of intact and homogenized sample and illustrates how the transfer model operates, comparing microspectroscopic data to the corresponding HTS-FTIR spectra for the same species. Attempts to calibrate directly in the image domain without applying the transfer were unsuccessful for both homogenized and intact biomass. This underscores the necessity of a microcalibration strategy with pixel-wise application of the transfer model.

For validation, we have applied microcalibration of lipid profiles and glucosamine content to microspectroscopic data of filamentous fungi. These oleaginous filamentous fungi serve as production organisms in modern biorefineries, yielding high-value lipids and coproducts such as chitin/chitosan, polyphosphates, and pigments.^14^ Their filamentous structure produces varying amounts, sizes, and types of lipid bodies under different growth conditions. This serves as an example of a bioprocess whose details we can better understand through spatially resolved quantitative analysis. In particular, better understanding of where different chemical compounds are synthesized within the fungi is of significant interest to both research and industry, as this knowledge could enable further process optimization.

To test the performance of our microcalibration model, we first confirm that the IR spatially resolved lipid concentration on average agrees with the GC measurements of the lipid content. We compare the mean pixel prediction of the microcalibrated images with the GC results of the same sample, expecting a fairly strong correlation. While significant intraimage variance is expected, it should average out fairly well to match the GC-measured values for sufficiently large images. We analyze the microspectroscopic images of both the homogenized and intact biomass.

To robustly evaluate the approach, we perform leave-one-out cross-validation, training one regression model for each of the 36 samples. Specifically, for each sample used for inference, the microcalibration model is trained using the measurements of the remaining 35 samples. We have also considered multitarget calibration models, where we predict e.g. total lipids, SFA and, PFA simultaneously, but this resulted in a slight drop in performance, so we present results from several single-target models.

Figure 3 shows that the average spatially resolved lipid concentration for homogenized biomass correlates strongly with GC with a coefficient of determination *R* ^2^ = 0.80. That is, on average the microcalibrated images account for 80% of the variability in total lipid content. We note that the homogenization seems to not work perfectly for a couple of samples, which, because we only have a single image-tile, leads to very large error-bars for those samples. As anticipated, for images of intact biomass, we observed a drop to *R* ^2^ = 0.72. The intraimage standard deviation is also significantly larger due to the heterogeneous nature of the cultivated fungal biomass, which includes both filaments and lipid bodies distributed throughout the image, which only covers a finite amount of biomass. We note for comparison that the regression model based on the macroscopic HTS-FTIR spectra, explained 84% of the variability in the total lipid content. This confirms that the microcalibration model retains much of the initial regression model’s predictive power. Certainly, a slight decrease in the coefficient of determination was to be expected, compared with predicting based on the HTS-FTIR spectra directly, since we have limited image sizes and there is inevitably a significant interimage variability. Nonetheless, the value of *R* ^2^ did not decrease too much for the microcalibration of the homogenized data implying that our quantitative model works well.

**Figure 3:**
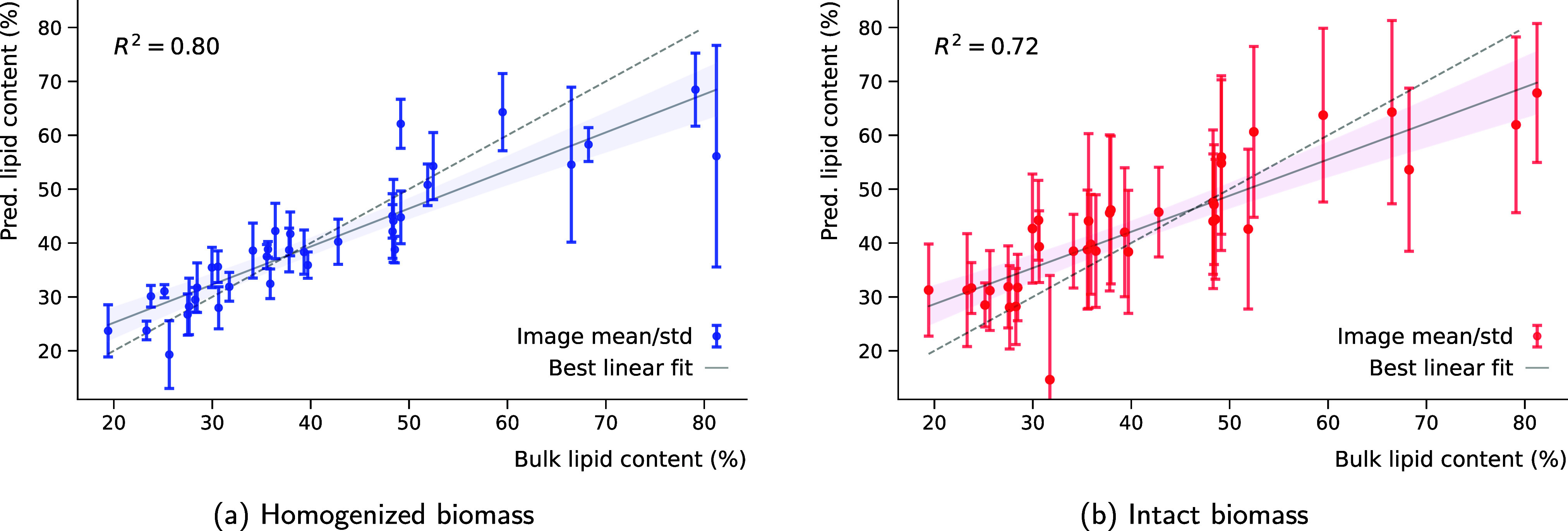
Predicted lipid amount in the microspectroscopic image and the lipid amount as given by GC for (a) homogenized and (b) intact biomass. The error-bars correspond to the image standard deviation. The predictions were all obtained through leave-one-out cross-validation, where independent microcalibration models were trained for each of the 36 samples. The best linear fit to the data is displayed as a gray line surrounded by the shaded region representing the 95% confidence interval. The dashed gray line signifies the line of perfect correlation between the bulk measured and average of the predicted image values of lipid content.

Generally, we observe higher accuracy for values of lipid content which are closer to the mean, and we see slightly lower accuracy for the fungal strains which are very good and very bad producers of lipids. This is because the training data are clustered in the 30–50% lipid content range, with few samples below 25% or above 55%. The standard calibration models built based on the macroscopic spectral data, have the same regression-toward-the-mean limitations. To alleviate this issue and make the models perform even better we could collect more training data with very low and high lipid content.

Furthermore, the distributions of the inferred lipid concentration in Figure 4 demonstrate that, while the average value closely align with the GC-predicted value, the intraimage variability is retained very well. Filamentous fungi like the ones studied here are known to produce lipid bodies which are essentially pure lipid droplets encapsulated by a thin cell wall, and they grow long hyphae which contain mostly polysaccharides and polyphosphates.^14^ For several samples where the image mean disagrees significantly with the GC results, we find a bimodal distribution of the pixel values, where the modes correspond to lipid droplets and hyphae. This indicates that the discrepancy with the bulk measurement stems from the finite-size image not perfectly capturing the different types of cultivated fungal biomass. This is more so the case for the species producing higher amounts of lipids such as *Umbelopsis vinacea* in Figure 4c,d which grow a large number of nearly pure lipid droplets, and less so for *Mucor racemosus* in Figure 4b which produce significantly less lipids.^14,34^

**Figure 4:**
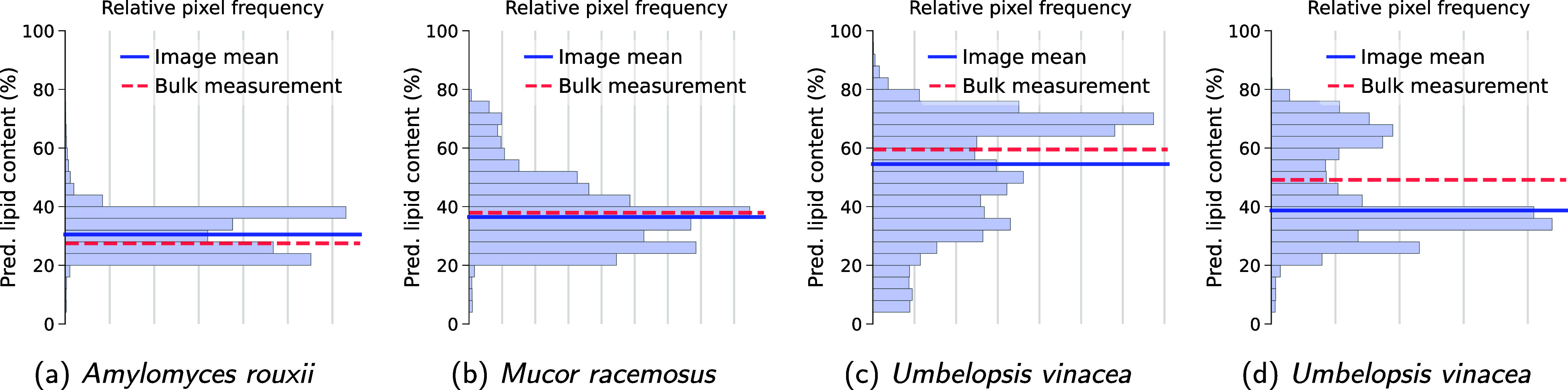
Histogram of the distribution of the predicted lipid content in pixels of images of intact (a) *Amylomyces rouxii* (with Ca supplementation and 0.5× the reference amount of P in medium), (b) *Mucor racemosus* (with Ca and 1× P), (c) *Umbelopsis vinacea* (without Ca and 4× P), (d) *Umbelopsis vinacea* (with Ca and 0.5× P). Horizontal lines give the mean value for the predicted lipid content in the image and the value given by GC. Empty pixels that do not cover any part of the sample have been excluded.

We find qualitative corroboration of our approach by comparing the predicted spatially resolved lipid profiles with existing literature.^14^ We leverage the existing knowledge and hypotheses about these well-studied oleaginous filamentous fungi to validate the biological and biochemical plausibility of our predictions. This is done to make sure that the models’ predictions of the spatially resolved lipid profiles are aligned with the fungi’s known biochemical behavior.

Figure 5 shows that the fungi have spherical regions which our model predicts to comprise 70–85% lipids as well as filamentous structures connecting these regions, which contain approximately 40% lipids. This aligns with the literature indicating that such filamentous fungi produce lipid droplets which are nearly pure lipid encapsulated by a thin cell wall. Pixels covering the lipid bodies inevitably also capture signals from the cell wall, so the predicted lipid content of 70–85% is consistent with expectations. Additionally, it is known that the filaments are fungal hyphae generally containing significantly less lipids,^14^ which supports our model’s prediction of a 40% lipid concentration.

**Figure 5:**
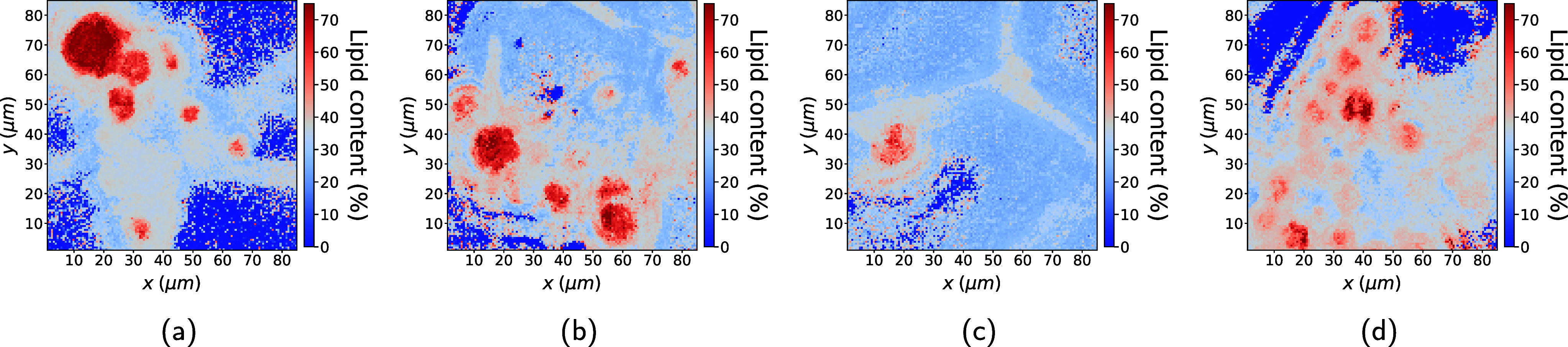
Qualitative validation of microcalibration applied to four strains of *Mucor circinelloides*. Images show the inferred spatial distribution of total lipid content, with color indicating lipid concentration. Empty pixels are retained in the visualization, as the model is trained to assign zero lipid content to regions lacking biological material.

Figure 6 further demonstrates a plausible distribution of saturated (SFA) and polyunsaturated (PFA) fatty acids, where the lipid droplets exhibit a higher proportion of SFAs, while PFA and SFA occur in approximately equal amounts in the hyphae and cell wall. This reflects the established tendency of filamentous fungi to store saturated fatty acids primarily in lipid droplets, with the remaining biomass maintaining a more balanced distribution of saturated and unsaturated fatty acids.^14^

**Figure 6:**
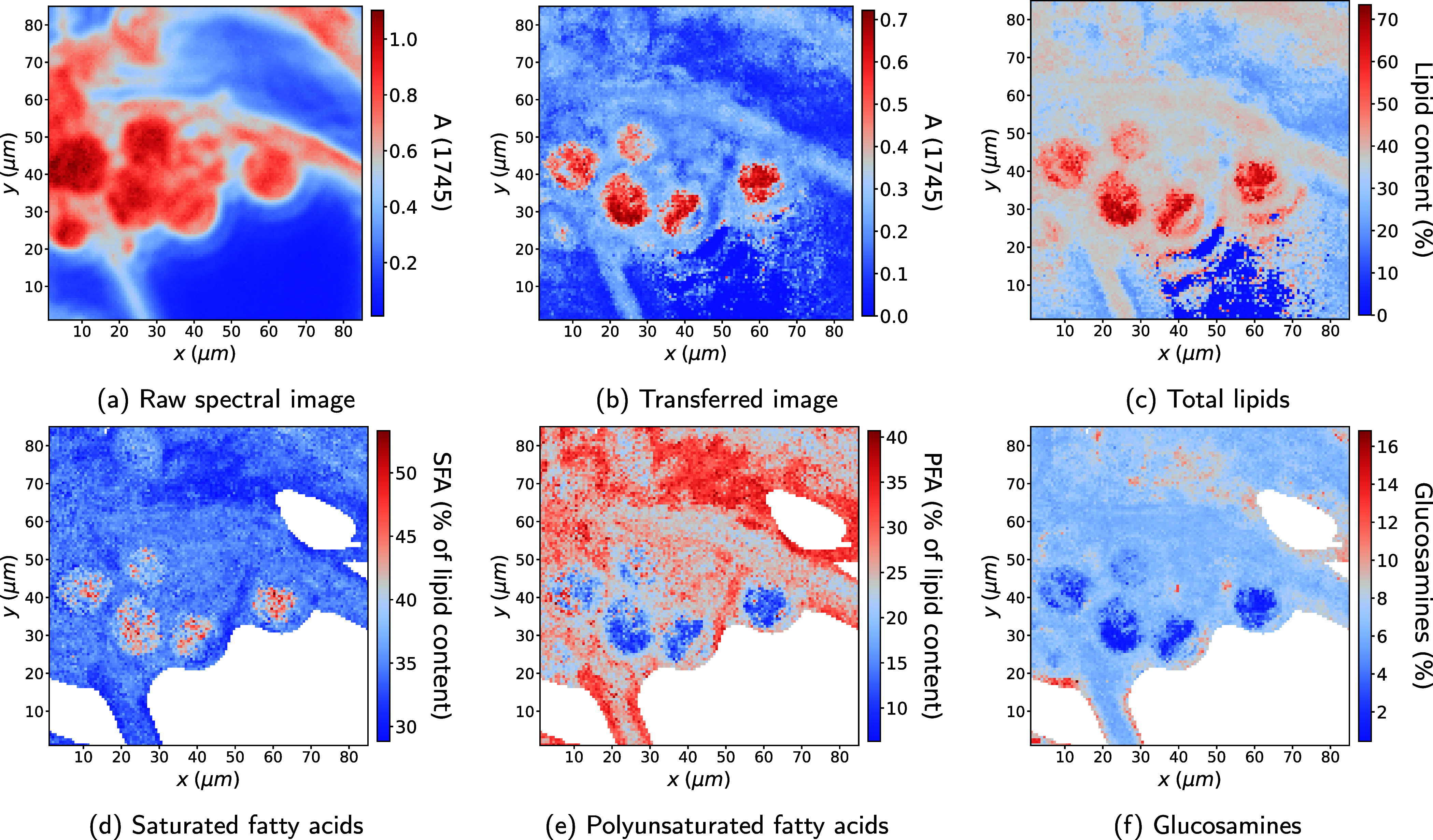
Microcalibration results for a single *Mucor circinelloides* sample for qualitative validation. (a) Raw microspectroscopic image and (b) transferred image, both at the ester peak at ν̃ = 1745 cm^–1^. Microcalibrated maps of (c) total lipid content, (d) saturated fatty acids (SFA), (e) polyunsaturated fatty acids (PFA), and (f) amount of glucosamines (GlcN). For SFA, PFA, and GlcN, pixels containing negligible biomass were masked out, since the calibration models predict the quantities as a percentage of biomass.

Moreover, the model predicts minimal glucosamine content in lipid droplets, with higher concentrations localized in the cell walls and filaments. This finding is fully supported by literature, as chitin and chitosan are key structural components of fungal cell walls.^14,34^

## Conclusions

We have demonstrated that quantitative label-free IR imaging is possible by applying our microcalibration approach, where we transfer conventional calibration of macroscopic HTS-FTIR spectra toward reference analysis to the imaging domain. This is done by handling the electrodynamic scattering on the intact samples and accounting for differences between macroscopic and microscopic measurements. We transfer the raw images so that each pixel spectrum resembles a macroscopic spectrum, allowing us to apply existing macroscopic calibration models. This allows for obtaining the two-dimensional distribution of fatty acid composition in intact biological samples in a nondestructive and label-free manner. Additionally, we showed that the approach can be used to infer spatially resolved glucosamine amounts. This eliminates the need for time-consuming, destructive, and expensive reference analyses and, more significantly, allows quantitative imaging-domain analysis of virtually any reference method, a capability that was previously unattainable. The method offers a simple and cost-effective tool for both research and industry to analyze sample features in the imaging domain.

We anticipate that the microcalibration model will transfer effectively across different types of microorganisms. Even when establishing the model from scratch is necessary, the effort required remains modest. The approach introduces minimal overhead beyond the standard workflow, which includes calibration using HTS-FTIR spectra and reference analyses, as well as IR image acquisition. Since biomass is already homogenized for HTS-FTIR measurements, the only additional steps needed are acquiring IR images of the homogenized biomass and training the transfer model. The latter requiring approximately 1 day and being readily automated.

While this study demonstrates the potential of the approach, future work will further evaluate its accuracy, spatial resolution, and performance for individual fatty acids through comparison with techniques such as mass spectrometry imaging. We also aim to investigate the microcalibration method’s applicability to a broader range of organisms and tissue types and to quantify the extent of retraining required in such cases. We will also investigate unifying the transfer and calibration models into a single model to streamline the approach, which could also allow for performing reliable spatially resolved uncertainty quantification.

## Data Availability

Data used in this study is stored under 10.5281/zenodo.16925484 and can be made available upon reasonable request. The code to perform microcalibration based on IR microspectroscopic data can be found at https://github.com/eirikama/microcalir.
